# Diagnostic Performance of Integrated ^18^ F FDG PETMR in the Diagnosis of Recurrent Foot Infection—Comparison with ^18^ F FDG PETCT and Conventional ^99m^ Tc MDP Bone Scan

**DOI:** 10.1055/s-0044-1800836

**Published:** 2024-12-09

**Authors:** Padma Subramanyam, Shanmuga Sundaram Palaniswamy

**Affiliations:** 1 Department of Nuclear Medicine & Molecular Imaging, Amrita Institute of Medical Sciences & Research Centre, Cochin, Kerala, India

**Keywords:** FDG PET, PETMR, foot infection, osteomyelitis, PETCT, MDP bone scan

## Abstract

**Introduction** Ulcerated foot is a forerunner for amputations among diabetics. Early detection of foot complications is imperative for guiding management; more so in recurrent foot infections.

**Purpose** The objective of this study was first, to determine the diagnostic performance of integrated Fluorodeoxyglucose (FDG) Positron emission tomography (PET) magnetic resonance (MR) in suspected soft tissue infections (STIs)/osteomyelitis (OM) in patients presenting with recurrent foot infections. Second, to compare regional [ ^18^ F] fluoro-2-deoxy-2-d-glucose ( ^18^ F FDG) PET Computed tomography (CT) and conventional three-phase ^99m^ Tc methylene diphosphonate (MDP) bone scan (BS) in this group of patients along with integrated PETMR.

**Materials and Methods** A total of 21 adult patients with suspected recurrent foot infections were prospectively enrolled from March 2020 until September 2023 in our tertiary care center. All patients were primarily referred for a regional PETMR (foot) study. We instituted a protocol to combine three-phase ^99m^ Tc MDP BS followed by PET imaging the next day (PETMR followed by PETCT). Images were correlated with patients' foot symptoms and clinical examination.

**Results** Diagnostic performance of ^18^ F FDG PETMR was superior compared with other two imaging modalities for STIs and OM. Using ^18^ F FDG PETCT, sensitivity, specificity, and accuracy for diagnosing soft tissue (ST) foot infections was 91, 71, and 79%, respectively, while for PET MR, it was 99.4, 100, and 98.6% versus 74.4, 31.2, and 62% for BS.

**Conclusion** Our study recommends the use of integrated ^18^ F FDG PETMR for podiatry-related problems, as it provides excellent ST demarcation and information on associated bone involvement, if any. It helps in accurately differentiating OM versus Charcot's foot; more so in surgically intervened or previously debrided foot when compared with the other two modalities. ^18^ F FDG PETMR clearly demarcates the depth and extent of surgery one must perform to get a reprieve from occult pockets of infection so as to attain a disease-free status. Given the paucity of evidence for integrated PETMR usage in foot-related indications, our small sample study highlights its superiority for clearly delineating and diagnosing various foot pathologies, infections, especially in the clinical setting of postsurgery/debrided foot.

## Introduction

Foot problems are common in an aging population due to weight-bearing effects and diabetes mellitus. 1 Ulcerated foot is a forerunner for amputations among diabetics. Early detection of foot complications is imperative, as it not only identifies the presence and extent of infection but also helps in guiding management and ensuring a higher clinical outcome. Osteomyelitis (OM) and Charcot's foot (CF) are commonly encountered in diabetic patients due to various factors. Approximately 20% of moderate-to-severe diabetic foot infections result in lower extremity amputations. 2 Identification of the site and extent of OM/surrounding soft tissue infections (STIs) help in assessing the extent of debridement and curtailing the disease process. Clinical differentiation between STI, CF, and OM is challenging. Depending on the presentation, foot OM can be classified as acute, subacute, or chronic type. Pain, fever, and raised inflammatory markers can occur and overlap infection and inflammatory conditions. The presence of ischemia, vasculopathy, and neuropathy further may lead to delayed wound healing. Such repeated infections lead to deformation of the foot further compounding the problem. 2 Based on the etiology, diabetic ulcers may be described as neuropathic, ischemic, or combined types. For the healing of long-standing nonhealing ulcers (NHUs), the underlying pathology and microbial growth have to be identified. Unless there is adequate control of blood sugar along with appropriate use of broad-spectrum antibiotics, infections cannot be controlled. At times, foot deformity may also need correction to avoid repeated infection and trauma.

Radiograph, Computed tomography (CT), and Magnetic resonance imaging (MRI) are used mainly for diagnosing foot problems. Although radiographs are inexpensive and widely available, early changes may be missed. It needs at least 30 to 50% bone loss for identification and interpretation of bone pathologies. However, it provides information on associated fractures, soft tissue (ST) swelling, edema, gas, and ulceration. CT proves to be a useful modality to detect early osseous erosion and to document the presence of sequestrum, foreign body, or gas formation but generally is less sensitive for the detection of bone infection. 3 In spite of this limitation, CT is preferred over conventional radiographs for assessment of the osseous structures, progressing infections such as any change in stage of OM (i.e., from acute to chronic) by characteristic bone changes. MRI remains to be the most sensitive and specific imaging modality for diagnosing OM, as it provides excellent ST contrast along with marrow signal alterations that may manifest even before bone lysis becomes apparent on radiography or CT. 3 Nuclear imaging such as a three-phase methylene diphosphonate (MDP) bone scan (BS) is found to be better than CT or radiographs alone being a physiological imaging procedure. Although it has a high degree of sensitivity, there is low specificity for BS, especially in bone infections. 4 Surgical intervention can produce false-positive MDP uptake further hindering its clinical relevance. With the wider availability of PET scanners, BS is increasingly being replaced by ^18^ F fluoro-2-deoxy-2-d-glucose ^18^ F FDG PETCT for OM evaluation. ^18^ F FDG PET CT has been found to be a useful imaging modality complementary to MRI, due to its higher specificity compared with MRI alone. The aim of this prospective study was to ascertain the value of PETMR in diagnosing foot infections and also to compare the diagnostic accuracy of these three commonly used modalities, that is, BS, PET CT, and PET MR in identifying various foot problems in diabetic and nondiabetic populations.

## Materials and Methods

This prospective study was undertaken from March 2020 until September 2023 in a tertiary care center after institutional ethical board clearance. Twenty-one adult patients with recurrent foot problems were enrolled.

### Inclusion Criteria

1. Patients (diabetic or nondiabetic) with one or more of the following presentations: foot pain, swelling, or ulcerations
2. > 4 outpatient visits in last 3 months for foot-related issues
3. Recent HbA1c report (range from 5 to > 7%)
4. Consent for undergoing all three imaging procedures
5. Previous surgical/medication history available
6. Only those patients whose last debridement was at least 6 weeks ago were included

### Exclusion Criteria

1. Children and pregnant women.
2. Patients with a recent history of surgical interventions/debridement of the foot were excluded.

The following patient information were collected: sociodemographic characteristics, including sex, age, height, weight, education level, occupation, disease-related information such as duration of diabetes, HbA1c, presence of diabetic peripheral neuropathy, prior foot infection, trauma, and surgical intervention, if any.

### Clinical Examination of Foot

Feet were clinically evaluated for swelling, pain, tenderness, and ulcers. Four patients had a history of surgical intervention, skin grafting/debridement. History of antibiotic coverage was elicited that included the drug combinations, dosage, duration, and date of its stoppage. Each foot was evaluated for the number of ulcerations, location, size, depth, shape of ulcer/s, surrounding inflammation, edema, exudate, past treatment, and duration of treatment. The margins of the ulcer were checked for callus formation, maceration, and erythema. The presence of erythema along with other signs such as tenderness and warmth were considered as corroborative markers for infection. The quality of the tissue (i.e., moist, granular, desiccated, necrotic, undermining, slough, eschar, or liquefied) was also noted. Note was also made for the presence of any sinus track or deep abscess on inspection and palpation of foot.

### Statistical Analysis

Statistical analysis was performed using SPSS (IBM Corp., Armonk, New York, United States) 24.0 software. Sensitivity, specificity, accuracy, and negative and positive predictive values of ^18^ F FDG PET CT, PET MR, and BS were calculated; 95% confidence interval of the mean was also obtained. The specificity and sensitivity of FDG positive lesions were correlated using microbiological studies and clinical follow-up as the gold standard. The Fisher's *p* -value was used to determine the statistical significance of differences in the accuracy of comparing all three modalities. Multivariate regression analysis was performed for assessing adequate glycemic status.

### Procedure

BS was done on day 1 followed by FDG PET on subsequent day. Glycemic status is important prior to FDG injection. Hence all patients were checked for their fasting glycemic status prior to ^18^ F FDG injection (dose of FDG injected was 0.1 mCi/kg body weight). Simultaneous PETMR imaging of feet was followed by PETCT as a single injection same day protocol. Intravenous (IV) contrast was reserved for PET MR studies, if necessary. PET CT was performed on Siemens Biograph Horizon 16 slice system, while PET MR was acquired using Siemens Healthcare Biograph mMR system (Erlangen, Germany) with body coil placed over feet.

### Imaging Protocol

#### Three-Phase Regional Bone Scan—Day 1

^99m^ Tc MDP was administered intravenously (antecubital vein) at a standard adult dose of 15 mCi. Immediate dynamic (vascular phase) foot images (128 × 128 matrix; 2 seconds/frame) were acquired for 60 seconds followed by ST phase static images (256 × 256 matrix; 500 kilo counts). Three hours later, the skeletal phase images of feet and ankles were acquired using a dual head variable angle Gamma Camera (GE NM 640 SPECT CT). SPECT CT images were later acquired at 25 seconds/frame for 360 degrees in a 64 × 64 matrix.

#### FDG PETMR—Day 2

On day 2, ^18^ F FDG PET study was conducted 45 to 60 minutes postinjection (PETMR was followed by PETCT). Two bed positions were acquired to include bilateral ankles and feet. Images were acquired on a Biograph mMR scanner having an axial field of view (FOV) of 25.8 cm, 65.6 cm ring diameter, a National Electrical Manufacturers Association (NEMA) specified spatial resolution near FOV center of 4.4 mm, and sensitivity near FOV center of 13,200 cps/MBq. FDG PET acquisition was extended to cover the duration of MRI acquisition, ranging from 10 to 20 minutes. Attenuation maps were also obtained by a four-tissue (air, ST, fat, and lung) Dixon-volume-interpolated mode. All attenuation maps were qualitatively examined visually during the scanning process. Acquired images were corrected for scatter, attenuation, point spread function, and time of flight and reconstructed in a 344 × 344 matrix with OSEM iterative reconstruction, three iterations and 21 subsets with a 4-mm Gaussian filter. Standard MRI sequences for foot were acquired: T1 turbo spin echo (TSE), T2 TSE Dixon, proton density TSE, and T2 with fat suppression by Short tau inversion recovery (STIR) sequences.

#### ^18^ F FDG PET CT

Feet images (including ankles) were acquired on the PETCT system. Images were acquired for 5 minutes using a 180 × 180 matrix at 3D collection mode for PET acquisition. No IV contrast was used for the CT study. The FOV for the PET CT scan was large, with a CT tube voltage of 130 kV, tube current of 115 mA with full rotation length, and interval of 3.260 mm. The scan speed was 17.50 mm/rotation with a pitch of 0.8. The CT images were acquired in a matrix of 512 × 512 with a window width of 350 and a detector 24 rows. Images were reconstructed using iterative reconstruction.

### Interpretation

#### BS Interpretation

Findings of increased vascularity, ST tracer uptake, and increased skeletal MDP tracer uptake in the involved bones/sites of ulceration of the foot were reported as OM. Patients diagnosed as inflamed CF showed focal/diffuse increased MDP uptake in the involved bones/joints in all three phases of BS. In those patients without bone involvement or ulceration, the diagnosis of cellulitis or STI was made. In all patients, number of hot spots in each bone/ joint was counted and tabulated.

### FDG PET Interpretation

#### Diagnosis of OM

Abnormal FDG uptake in the foot was characterized as focal or diffuse. The number of sites, location, and extent of FDG uptake were noted along the bone/s or ST. Clinical examination findings including physical inspection of the ulcer along with corresponding MR or CT findings were correlated. Patients with no associated bony involvement were reported as STI. Each modality images were interpreted separately by senior nuclear medicine physicians with more than 20 years' experience, blinded to patient details.

Following visual and quantitative parameters were checked in all images as follows:

1. Visual PET findings:
  (a) STI and its extent (Grade 1: ST immediately surrounding the involved bone, Grade 2: limited to the same region, i.e., forefoot, midfoot, or hindfoot, Grade 3: involvement of the adjacent or subsequent region/ankle, Grade 4: surrounding joints involved). In patients with only ST involvement (no bony involvement), based on site involved, the region was considered as forefoot, mid-foot, and hindfoot involvement.
  (b) Marrow involvement
  (c) Cortical disruption
  (d) Sequestra, if any
  (e) Single or multiple ulcers, its location and extent
  (f) Presence of fistula (extension to skin or not).
2. Quantitative PET parameters:
  (a) Standardized uptake value, maximum (SUV) max: SUV max (based on body weight) of each lesion was obtained and tabulated. Any lesion with an SUV max of 2.5 and above were considered abnormal. Findings were also correlated clinically with (A) location and number of ulcer/s, (B) visualization of any external fistula/sinus tract, (C) foot swelling (forefoot, mid-foot, or hind foot), (D) ankle swelling, and (E) previous debridement site, extent.
  (b) Additional quantitation such as target-to-background ratio (TBR) on MIP images was also obtained to discern FDG uptake in pathological STI versus postoperative inflammatory setting in the debrided foot. TBR was calculated in all patients using the following methodology: lesional SUV max divided by the average SUV in an internal reference region close to the lesion with a visually normal FDG uptake, normal appearance on CT or MR images, respectively.

### Diagnosis of CF

On visual analysis, FDG uptake in bones involved due to CF may be variable. Generally, diffuse low-grade FDG uptake along the involved bones and joints was interpreted as diagnostic for CF. Such sites were found positive on CT/MRI. Clear demarcation of FDG uptake pattern was observed with SUV max values in normal joints (discernible from CF-affected joints). Unaffected joints in the ipsilateral or contralateral foot showed SUV max, ranging from 1 to 1.5, equivalent to background. As bone uptake on FDG PET was not very high, TBR was nearly equivalent to background in CF patients (range 1–1.3).

### Diagnosis of Cellulitis

Patients with only diffuse FDG uptake (SUV max > 2.0) with no bony involvement were categorized as cellulitis.

End points of the study (1) bone/ST culture and sensitivity in patients referred with a suspicion of OM/cellulitis/CF and (2) clinical improvement at least 6 months after adequate management were considered as end points for diagnostic correlation.

## Results

A total of 21 patients, majority being males (M:F = 14:7) with podiatry problems were included. 17 patients had diabetes mellitus with > 10 years' duration. Range of HBA1c in our patients was 5.7 to > 7%. Clinical, demographic, and imaging findings are shown in Table 1 . 8 out of 21 patients (3.8%) had prior surgical intervention/debridement of wounds (4 patients had more than once). Ulcer diameter ranged from 2 mm to 2 cm in size on MR. NHUs were noted in the following locations on clinical examination: tarsal bones (7 patients), phalanges (7 patients) followed by metatarsal bones in 6 patients and calcaneum/hindfoot in 3 patients. Table 2 demonstrates the final diagnosis obtained by the various imaging modalities.

**Table 1:** Clinical, demographic, and imaging findings

Pt	Age/sex	Episodes of pain/infection	Diabetic status	Surgical; intervention (+ once debrided/+ + twice)	No. of bone: ST lesions on PET	MR; CT findings	Final diagnosis
1	56/M	Pain, exudate, induration, ulcer	DM	+ +	Three bone lesions + extensive ST involvement	Three bone lesions; no CT bone lesion, STI noted	OM + ST infection
2	54/M	Pain, swelling, ulcer	DM	No	Four bone lesions with additional ST lesions	Four bone lesions with extensive edema; two MT shaft lesions on CT	OM of MTs with extension
3	65/M	Old calcaneal OM Pain, exudate, induration, ulcer	DM	+ + with skin grafting	No residual calcaneal bone Extensive ST involved	T2 hyperintensities in right plantar aspect ST, no lesion on CT but STI +	Old calcaneal OM with residual STI
4	47/M	Pain, swelling, ulcer left third toe	DM	+	Tarsal bones +/MTP joints, MT bone lesion + surrounding ST involvement on MR	MR images of right foot show periarticular high T2W signal with enhancement around TMT joints. The typical location, coupled with absence of other secondary ST signs of infection indicated CF with OM left third phalanx. CT-wise erosion of TMT joints	CF right foot with OM left third toe
5	36/F	Swelling, tenderness, warmth	No DM	No	Cellulitis	Increased reticulation of subcutaneous fat of lower leg and foot, with corresponding high T2W signal and enhancement—cellulitis. No CT lesion	Cellulitis
6	51/F	Pain, swelling, ulcer	DM	No	Left first MT head involved, ST +	T1 hypointense, T2/STIR hyperintensity left first MT head	OM
7	43/F	Pain, swelling ankle	No DM	No	Achilles tendinitis	Edema along tendon on MR	Achilles tendinitis
8	56/M	Pain with ulcer foot		+	Subtalar joint; phalanx involved	Subtalar joint on MR, no CT finding	OM phalanx
9	39/M	Ankle swelling and pain	No DM	No	No significant FDG avidity	Deformed foot	CF
10	67/M	Pain, swelling ankle, ulcer	DM	+ + + with lot of slough	Three bone lesions with ST uptake	One bone lesion on MR low-T1W signal in inferior and medial part of the talus with high signal on postcontrast and T2W images—OM	OM
11	77/F	Pinprick injury 3 months back with non healing ulcer	DM	No	Two bone lesion	Low T1-W and high T2-W marrow signals in MT head with associated cortical ill-definition, suggest OM	OM
12	56/F	Ankle swelling ? synovitis	No DM	No	Cellulitis	Cellulitis	Cellulitis
13	27/M	Fever, heel pain	DM	No	None	None	Achilles tendinitis left
14	35/M	Pain, exudate, induration, NHU	DM	+	Calcaneum: ST involvement	Calcaneum + ST involvement	OM
15	66/M	Ulcer phalanx	DM	No	Two bone lesions	OM two phalanges	OM
16	72/M	Pain, swelling foot	DM	No	MTP joints	MTP joints	CF
17	62/M	Pain, swelling foot suspected CF	DM	No	Mid-tarsal and MPT joints	Mid-tarsal and MPT joints	Bilateral CF
18	48/M	Heel pain	DM	No	FDG uptake at insertion of plantar fascia	Thickened plantar fascia on MR	Plantar fasciitis
19	55/F	Pain foot and ankle region	DM	No	Tarsal bones	Partial destruction of cuboid and medial cuneiform	CF, OM
20	48/F	Nonhealing callus ulcer	DM	+	MT head	“Tram-track” pattern of a sinus tract of plantar forefoot. Sinus tract extends to a subcutaneous abscess, adjacent to base of first MT with overlying skin ulcer. Adjacent bone marrow enhances, suggesting OM	OM
21	64/M	Pain, swelling, nonhealing ulcer foot	DM	+ +	Tarsal bones	Edema and joints involved on MR, with superadded ST and sinus tract. No OM on CT/MR	OM + CF

Abbreviations: CF, Charcot's foot; CT, computed tomography; DM, diabetes mellitus; FDG, fluorodeoxyglucose; MR, magnetic resonance; MT, metatarsal; MTP, metatarsophalangeal; NHU, nonhealing ulcer; OM, osteomyelitis; PET, positron emission tomography; ST, soft tissue; STI, soft tissue infection; STIR, short tau inversion recovery; T2W, T2-weighted; TMT, tarsometatarsal.

**Table 2:** Visual and quantitative parameters in 21 patients using all three modalities separately

Final diagnosis	PET	MR	CT	MDP BS
OM	12	12	7	18 a
STI	14	20	3	2
CF	6	4	9	9
Combination	4	4	–	9
Other variables noted				
Sequestra	None	None	None	None
Ulcer	12	15	12	5
Fistula/sinus tracts: open (blind)	0	5 (2)	0	2
Debridement/surgery	4	4	4	None
SUV max range (g/mL)	2.6–7.8	2.1–12.3	Not applicable	Not applicable
FDG TBR (mean)	4.9	9.4	Not applicable	Not applicable

Abbreviations: BS, bone scan; CF, Charcot's foot; CT, computed tomography; FDG, fluorodeoxyglucose; MDP, methylene diphosphonate; MR, magnetic resonance; OM, osteomyelitis; PET, positron emission tomography; STI, soft tissue infection; SUV max, standardized uptake value, maximum; TBR, target-to-background ratio.

a Eight of the sites were false positive on culture.

### Bony Lesions

**Fig. 1:**
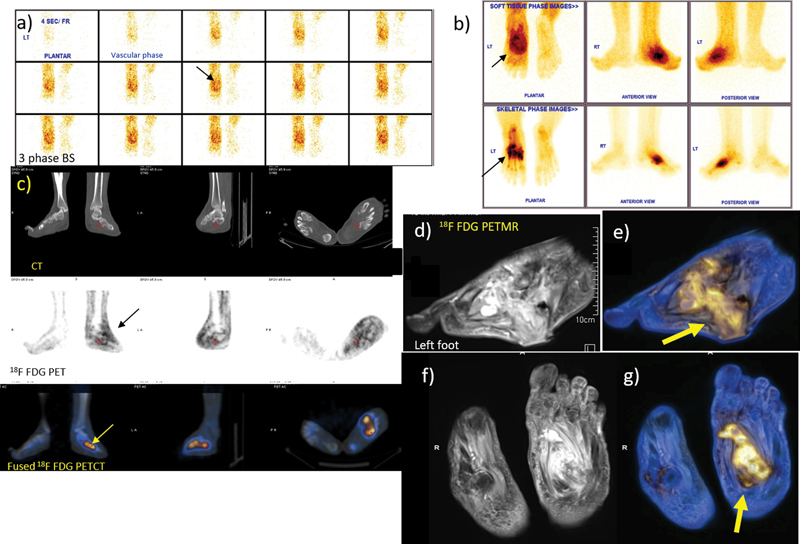
A 64-year-old man with NHU and sinus along plantar aspect of left foot, findings suggesting OM, surrounding soft tissue infection and CF (left foot). Three-phase ^99m^ Tc MDP bone scan: (a) Vascular phase images of feet. (b) Soft tissue and skeletal phase images of feet (arrow) showing increased vascularity at left mid-tarsal region. Soft tissue and skeletal phase images show intense MDP uptake in left tarsal bones suggestive of Charcot's foot, (c) ^18^ F FDG PET CT feet shows diffuse subtle FDG uptake in soft tissue of left foot with subtle mid-tarsal bone involvement. ^18^ F FDG PET MR: (d) Sag T2W MR, (e) fused PET MR images, (f) coronal T2W (g) fused coronal PET MR images showing clear delineation of bones, soft tissue involved (thick arrow). Thus, bone scan reveals an inflamed left mid-foot Charcot's arthropathy. FDG PET reveals superadded STI and osteomyelitis (SUV max 4.5) (arrow) proven by culture. Small ulcer on plantar aspect of mid-foot has minimal deep collection and tracking (arrow). However, MR shows no obvious signs of bone involvement (patient no. 21). CT, computed tomography; FDG, fluorodeoxyglucose; ^18^ F FDG, [ ^18^ F] fluoro-2-deoxy-2-d-glucose; MDP, methylene diphosphonate; MR, magnetic resonance; NHU, nonhealing ulcer; PET, positron emission tomography; STI, soft tissue infection; T2W, T2-weighted.

**Fig. 2:**
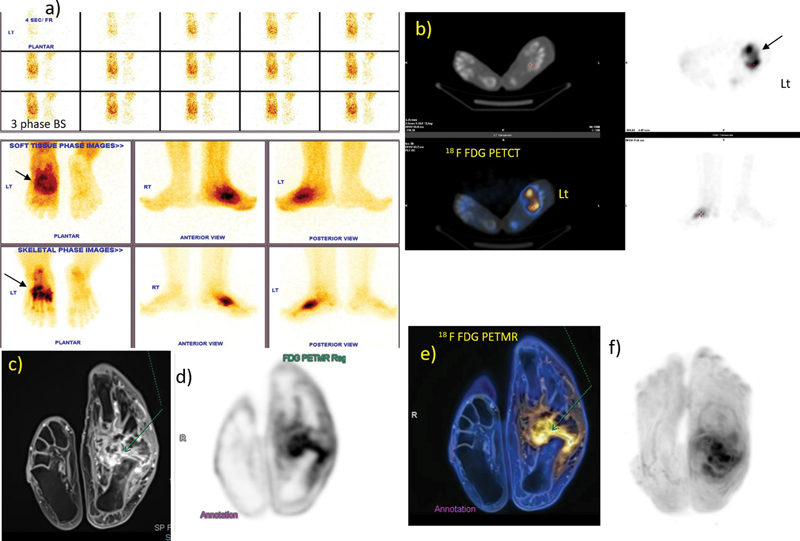
(a) Three-phase ^99m^ Tc MDP bone scan showing inflamed left Charcot's arthropathy, (b) ^18^ F FDG PET CT:transaxial CT and fused images of feet showing no obvious bony changes on CT with increased FDG uptake in left midtarsal bones, (c–f) ^18^ F FDG PETMR shows small ulcer on plantar aspect of left mid-foot with subtle deep collection evident on PET MR with associated diffuse synovial thickening and enhancement (arrow). CT, computed tomography; ^18^ F FDG, [ ^18^ F] fluoro-2-deoxy-2-d-glucose; MDP, methylene diphosphonate; MR, magnetic resonance; PET, positron emission tomography.

**Fig. 3:**
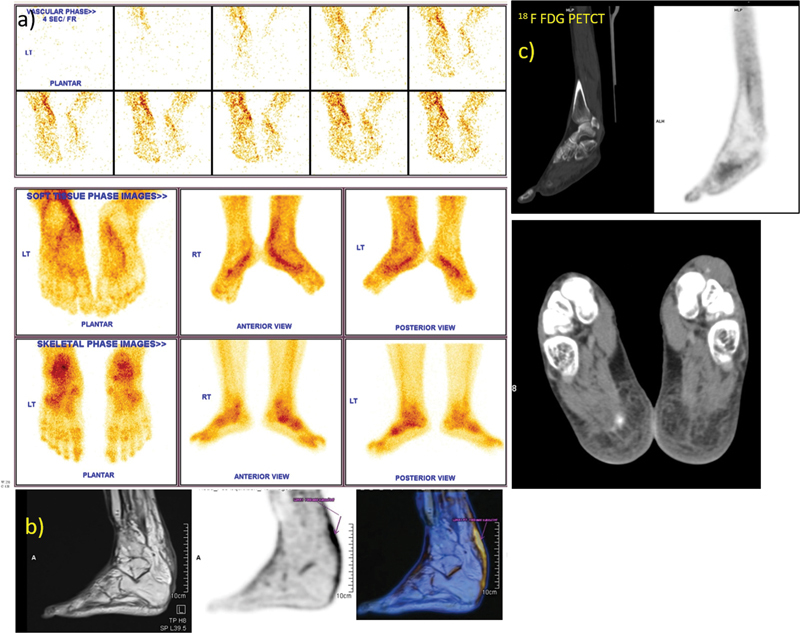
Left heel pain: (a) ^99m^ Tc MDP three-phase bone scan of feet, (b) ^18^ F FDG PET MR; T2W sag MR, FDG PET sag, and fused PET MR images showing left plantar fasciitis. PET MR shows linear FDG uptake corresponding to the peritendinous contrast enhancement in MR along left tibialis posterior, flexor digitorum longus, and flexor hallucis longus tendons, suggestive of tenosynovitis (patient no. 7), (c) ^18^ F FDG PETCT images, sagittal and transaxial views showing no increased FDG soft tissue uptake/bone involvement in feet. CT, computed tomography; FDG, fluorodeoxyglucose; ^18^ F FDG, [ ^18^ F] fluoro-2-deoxy-2-d-glucose; MDP, methylene diphosphonate; MR, magnetic resonance; PET, positron emission tomography; sag, sagittal; T2W, T2-weighted.

OM was diagnosed in 12 patients by FDG PET and various MR sequences independently. Although 18 sites of suspected OM were identified on BS, 8 of them were false positive on culture. Cortical disruptions/bone involvement in OM and CF cases were highest on PET MR. The number of culture-proven FDG-positive bony lesions on FDG PET MR was higher than PET CT and BS. FDG PET was not useful for CF evaluation, as most of them were negative. MR was also unable to identify all bony sites of CF unlike MDP BS/PET CT ( Table 2 ). CF was clearly demarcated on MDP BS and PET CT, but sites of occult trauma overlapped the diagnosis in three patients. Nine additional bony lesions identified on PET MR proved to be OM on culture ( Fig. 1 ). ST and bony cortical disruptions were clearly demarcated on each imaging with corresponding FDG/MDP uptake. Six patients were diagnosed with CF ( Fig. 2 ), one of them had bilateral involvement, two had cellulitis, two demonstrated features suggesting Achilles tendinitis ( Fig. 3 ), and one had plantar fasciitis on PET and MDP study.

### Soft Tissue Infection / Inflammation

14 patients were diagnosed as STI ON PETMR. Based on FDG uptake they were categorized as Grade 1 to 4 STI ( 3 : 3 : 5: 3 patients respectively). FDG avidity on PET MR clearly highlighted 11 additional sites of infection when compared with PETCT. MDP uptake in ST sites were unreliable and largely nonspecific. In patients with CF, the uptake pattern of FDG was largely poor or variable when compared with background activity (subtle diffuse increased FDG uptake). 2 patients had coexisting OM and CF proven on culture.

### Marrow Involvement

Marrow involvement was reported in 2 patients on MR with no corresponding FDG uptake suggesting edema. MRI (both T1 and T2w sequences) showed a larger extent of the edema in a few patients with OM when compared with FDG PET. In another 2 patients, edema and ST interpretation at three sites were diagnostically a challenge but finally were reported positive for infection on MR. However, these three sites showed no obvious FDG uptake, and the culture was negative for infection.

### Sinus/Fistula

Five open and two blind sinus/fistulous tracts were visualized clearly on MR when compared with CT and BS.

### Glycemic Status

As lesion detection depends on the degree of FDG avidity, we also correlated the glycemic status with SUV. The multivariate regression analysis adjusted for other factors affecting SUV showed no relationship between the patients' glycemic state and the degree of ^18^ F FDG avidity in infected sites ( *p* = 0.178) ( Table 3 ).

**Table 3:** Multivariate regression analysis for glycemic status and SUV max

Parameter	Foot FDG PET imaging CE (95% CI)	*p* -Value
Male	0.000 (−0.007, 0.007)	0.990
Female	0.000 (−0.004, 0.005)	0.890
BMI (kg/m ^2^ )	0.014 (0.013, 0.015)	<0.001
Dose injected (mCi)	−0.001 (−0.002, 0.001)	0.536
Glycemic status (g/dL) with respect to FDG uptake (SUV max) (cutoff fasting blood sugar 150 g/dL)	0.019	0.178
< 150 −0.007 (−0.017, 0.004) > 150 −0.015 (−0.059, 0.029)		

Abbreviations: BMI, body mass index; CE, coefficient estimate; CI, confidence interval; FDG, fluorodeoxyglucose; PET, positron emission tomography; SUV max,  standardized uptake value.

Notes: All multivariate models were adjusted for sex, BMI, activity injected, and fasting plasma glucose level.

A list of patients diagnosed with OM, CF, and a combination of podiatry-related pathologies are given in Table 2 . FDG PET showed either focal/diffuse increased uptake in all infected sites with a mean SUV max of 5.7 (range, 2.6–7.8 on PET CT and higher range on PET MR, i.e., 2.1–12.3, respectively) for both osseous and ST sites of infection. The ratio of SUV max measured with PETMR compared with that measured with PETCT was close to 1 (range, 0.67–1.7). TBR was calculated from the MIP/planar MDP image by drawing regions of interest on both calves, feet, and uninvolved limb/thigh (as background). TBR values ranged from 3.8 to 7.7. TBR was highest for OM cases (mean 4.9 on PET CT and 9.4 on PET MR), being close to two times higher than PET CT TBR followed by BS (range, 1.3–1.8). Our study showed that the diagnostic performance for identifying STI was highest with integrated PET MR followed by OM ( Table 4 ).

**Table 4:** Analyzing performance characteristics of multimodality imaging (PETMR, PETCT bone scan) in diagnosing STI and OM

	Sensitivity	95% CI	Specificity	95% CI	Accuracy	NPV	PPV
Performance characteristics of patients undergoing multimodality imaging for STIs							
FDG PET CT	90.6	69.31–93.85%	70.8%	41–88.5%	79.1%	90.3%	72. 3%
FDG PET MR	99.45%	87.16–99.88%	100%	87.2–100.00%	98.62%	95.24%	100%
BS	74.4%	49.5–78.2%	31.2%	7.7–76.96%	62%	56.0%	69.5%
Performance characteristics of patients undergoing multimodality imaging for OM							
FDG PET CT	94.12%	71.31–99.85%	73.68%	48.80–90.85%	83.33%	93.33%	76.19%
FDG PET MR	95.45%	77.16–99.88%	100%	83.16–100.00%	97.62%	95.24%	100%
BS	84.62%	54.44–98.08%	28.57%	3.67–70.96%	65%	50.0%	68.75%

Abbreviations: BS, bone scan; CI, confidence interval; CT, computed tomography; FDG, fluorodeoxyglucose; MR, magnetic resonance; NPV, negative predictive value; OM, osteomyelitis; PET, positron emission tomography; PPV, positive predictive value; STI, soft tissue infection.

Microbiologic cultures for ^18^ F FDG avid ulcers ( *n* = 15) were positive for *Staphylococcus* / *E. coli* in our series. Antibiotics coverage was optimized based on culture sensitivity studies. Surgeons were provided with the fused PET MR images to plan the margin resectability and depth of debridement and ST clearance that needs to done. No relapses were noted within a span of 3 months' posttreatment. 8 patients needed prolonged medical management (> 3 months).

## Discussion

Foot, especially in the elderly, is prone to infection, inflammation, and joint pathologies. Each of these foot pathologies if left unnoticed/untreated may limit mobility. With advancing age and alteration in foot biomechanics combined with diabetic complications such as neuropathy, vasculopathy, and metabolic changes, foot ulcerations may develop. 5 They progress to OM and deep STIs much before clinical attention is sought. In pre-PET era, BS and 67Ga citrate imaging were used to study OM and CF. 6 Availability of ^18^ F FDG and better instrumentation, imaging has become easier with high sensitivity and better specificity for foot-related complications. 7 Based on the immunosuppressed state of the individual and the virulence of microorganisms growing in the foot ulcers, OM may be debilitating and may take a long time to heal. Clinically, it may be possible to suspect infection, but accurate diagnosis of occult sites is possible only by choosing the right imaging technique.

Many reports suggest that MRI and ^18^ F FDG PETCT are both valuable in diagnosing OM. However there is no single study showing head-to-head comparison of these two modalities with simultaneous PETMR for OM/CF diagnosis in the literature. The number of bony lesions and ST demarcation was best with PET in our study which was supplanted by MR anatomical delineation. Our PET findings are supported by a study published by Yuh et al. 8 They showed a high sensitivity and specificity for FDG PET studies with a significant difference from MDP BS in OM detection. Abdel Razek and Samir in their study 9 showed MRI as the preferred imaging modality for diabetic foot evaluation and detecting OM (77–100% sensitivity and 80–100% specificity). Our study highlights the highest diagnostic performance including specificity and positive predictive value in diagnosing each foot disorder when compared with the previous citations in the literature. 9

While comparing all the three nuclear imaging techniques, anatomical details of bones and STs were best obtained in our study from MR sequences, while CT provided details on cortical disruption and fractures. Advantages of FDG PET CT observed in our study were the shorter imaging time and better evaluation of lytic/sclerotic lesions. BS is most widely available, cost effective, easy to perform and interpret, but lacks specificity. The highest sensitivity and specificity for OM and STI as a “single stop shop” can be obtained only from simultaneous PETMR especially in patients with recurrent foot problems. Guidance to delve into the affected surgical planes, sinus tracts, deep collections, and joint spaces were clearly demarcated by PET MR and not by PET CT/MDP scans. This was crucial to clear occult residual infective pockets and avoid recurrence of infection.

While considering OM and CF patients, the pattern of ^18^ F FDG uptake in both these disease entities was found variable in our study, which is similar to the literature reports. 9 Our study revealed that FDG PET had a high negative predictive value in ruling out OM in such patients. We found focal intense FDG uptake in OM and acutely inflamed CF with ongoing infection. Burnt out/smoldering CF (i.e., bone deformity with inactive disease) showed subtle diffuse FDG uptake in surrounding bones and ST. 8 Thus, visual interpretation of ^18^ F FDG PET can be reliably used in the differentiation of OM versus CF. We additionally observed a higher SUV max and TBR in infected sites in PET MR when compared with PET CT. Patients with associated fracture/OM of the tarsal or metatarsal bones in CF also demonstrated FDG uptake.

PET is highly sensitive for identifying infection especially on PET MR due to higher resolution, longer imaging times and better count statics. Findings are further exemplified when performed under strict glycemic control as recommended in various studies. 10 11 Due to its excellent spatial resolution, even a small ulcer (2 mm) with subtle FDG avidity was easily identified on PETMR. The SUV max in one such patient was 2.4 with culture positivity for infection. Identifying and treating these small lesions can help in the early control of disease.

We also found that the upper limit of SUV max in PET MR was slightly higher than PET CT which is explained by the longer acquisition times. TBR values offer an additional quantitative parameter that can reliably detect a lesion as demonstrated in a study by Hulsen et al. 12 TBR was not greatly different when compared with SUV max in our study. Thus, it may be a useful adjunct in patients where the glycemic status is not optimum, but PET imaging has to be performed due to coexisting infective conditions. Given the paucity of evidence for integrated PETMR usage in foot disorders, our study is incremental in recommending its superiority for foot evaluation especially in postsurgical/debrided foot settings.

## Limitations of Our Study

The limitations of our study were (1) small sample size, (2) we were unable to enroll patients with OM, STI, or CF separately, (3) compliance and consent to undergo all three investigations with coexisting foot pathologies was a problem, especially in diabetic patients with neuropathy, and (4) cost factor.

## Conclusion

Integrated PETMR was found to be invaluable in identifying STIs and OM when compared with PET CT and conventional three-phase MDP BS especially in patients with repeated foot problems and debridement. Guidance to delve into the affected surgical planes, sinus tracts, deep abscess collections, and joint spaces was clearly demarcated by ^18^ F FDG PET MR and not by ^18^ F FDG PET CT/MDP scans. The accurate delineation of STI/bone involvement in podiatry practice is exemplified only by a combined ^18^ F FDG PET MRI. This is crucial to clear occult residual infective pockets and avoid the recurrence of foot infection.
